# Sleep Quality Mediates the Association Between Cerebral Small Vessel Disease Burden and Frailty: A Community-Based Study

**DOI:** 10.3389/fnagi.2021.751369

**Published:** 2021-10-20

**Authors:** Jing Zhao, Wensheng Qu, Xirui Zhou, Yinping Guo, Yi Zhang, Lingshan Wu, Zhiyuan Yu, Hao Huang, Xiang Luo

**Affiliations:** Department of Neurology, Tongji Hospital, Tongji Medical College, Huazhong University of Science and Technology, Wuhan, China

**Keywords:** cerebral small vessel disease, burden, frailty, sleep quality, mediation effect

## Abstract

**Background:** Physical frailty is a common problem among older adults which usually leads to adverse health outcomes. The imaging markers of cerebral small vessel disease (CSVD) are associated with frailty, but the underlying mechanisms remain unclear. The present study aimed to investigate the mediating role of sleep quality in the relationship between CSVD burden and frailty.

**Methods:** We performed a cross-sectional study and enrolled community residents. Frailty and sleep quality were measured using the Fried frailty phenotype and the Pittsburgh Sleep Quality Index (PSQI), respectively. A multivariate linear regression analysis and a Bootstrap analysis were performed to examine the association among the key variables and the mediating role of sleep quality.

**Results:** Of the 726 participants (mean age: 65.5 ± 6.5 years, 59.8% female), the numbers (percentages) of the frail, prefrail, and robust residents were 49 (6.7%), 310 (42.7%), and 367 (50.6%), respectively. After adjusting for covariates, the CSVD burden and PSQI score were significantly associated with the frailty score. In addition, sleep quality played a partial mediating role in the association between CSVD burden and physical frailty. The mediating effect was 21.9%.

**Conclusion:** The present study suggests that sleep quality is a mediator of this association between CSVD and frailty in community-dwelling older adults. Improving sleep quality might be helpful to mitigate the risk of frailty in CSVD patients.

## Introduction

Frailty is a clinical condition of increased vulnerability to physiological stressors due to a loss of homeostatic reserves. It is a major public health problem since it can lead to adverse events such as falls, disabilities, hospitalizations, and death (Fried et al., 2001). Frailty is a geriatric syndrome associated with aging (Darvin et al., 2014). With an accelerated aging population worldwide, and especially in China where the proportion of people aged 60 and over was 17.9% at the end of 2018 Jing et al. (2020) and is estimated to increase to approximately 30% by 2050 (Liu et al., 2017), the burden of frailty will increase rapidly. Therefore, it is urgent to find methods that can mitigate the risk of frailty.

In addition to having an increased risk of frailty, the older adults also have a higher risk of cerebral small vessel disease (CSVD) (de Leeuw et al., 2001). CSVD is a progressive syndrome with clinical, neuroimaging, and neuropathologic presentations related to the vessels of the brain (Wardlaw et al., 2019). The principal imaging markers of CSVD include recent small subcortical infarcts, lacunes, white matter hyperintensities (WMH), enlarged perivascular spaces (EPVS), cerebral microbleeds (CMBs), and atrophy (Wardlaw et al., 2013). Although several previous studies have demonstrated the associations between frailty and the neuroimaging markers of CSVD (Siejka et al., 2018, 2020; Kant et al., 2019; Maltais et al., 2019; Jordan et al., 2020), the underlying mechanisms of this relationship remain unclear.

Poor sleep is a common health problem among the older population (Phillips and Ancoli-Israel, 2001). Both frailty and CSVD are associated with sleep disorders (Del Brutto et al., 2015; Shih et al., 2020), but the complex relationships between CSVD, sleep quality, and frailty remain to be explored. In the present study, we hypothesized that the severe CSVD burden could be related to a higher risk of frailty, and this association might be mediated by poor sleep quality. A successful mediation could mean that the risk of frailty in patients with CSVD might occur due to sleep disorders. Our investigation could thus lead to strategies to prevent frailty.

## Materials and Methods

### Participants

The Tongji CSVD study is an ongoing community-based study in elderly residents in Wuhan, China. Five communities were randomly selected from Wuhan. All of the eligible residents were aged 55 years or older and living in the five communities that were invited to participate in our study. The exclusion criteria were: inability to live unaided; life expectance is less than 1 year; with a definitive processing central nervous system (CNS) disease like Alzheimer’s disease and Parkinson’s disease; with psychiatric diseases (e.g., major depression, schizophrenia); with acute diseases like stroke, trauma and infection during the past 3 months; any contraindication to magnetic resonance imaging (MRI); and, failure to communicate and complete an interview due to any reasons.

This study was approved by the Ethics Committee of Tongji Hospital, Tongji Medical College, Huazhong University of Science and Technology (No. S105). All participants provided written informed consent.

### Physical Frailty Assessment

Physical frailty status was assessed using Fried’s criteria, which consists of five items: unintentional weight loss, slowness, weakness, low activity, and exhaustion (Supplementary Table 1; Fried et al., 2001). Participants were classified as robust (no frail component), prefrailty (one or two frail components), or frailty (more than two frail components).

### Sleep Quality Assessment

Sleep quality over the past month was measured using the Pittsburgh Sleep Quality Index (PSQI), a questionnaire based on self-reported experiences. The PSQI consists of 19 items and ranges from 0 to 21. A global PSQI score of greater than seven indicates poor sleep quality (Buysse et al., 1989).

### Covariate Assessment

Information regarding age, sex, height, weight, education level, smoking, and alcohol drinking was recorded in a standardized questionnaire. The self-reported medical history, including hypertension, stroke, coronary heart disease, diabetes, hyperlipidemia, kidney disease, arthritis, migraine, or other medical issues, was also documented. Depressive symptoms were measured with the Hamilton Depression Scale (HAMD). To avoid circularity, sleep items were excluded when calculating the HAMD score.

### Brain Magnetic Resonance Imaging Acquisition

Brain MRI was obtained using a single 3T MRI scanner (United Imaging, Shanghai, China). Following predefined standardized protocols, the MRI included at least five sequences: T1-weighted, T2-weighted, fluid-attenuated inversion recovery (FLAIR), diffusion-weighted image (DWI), and susceptibility-weighted imaging (SWI) (see the Supplementary Table 2 for detailed MRI protocols).

### Cerebral Small Vessel Disease Total Burden Score Assessment

Two trained radiologists, who were blinded to all the clinical data, rated the neuroimaging markers of CSVD based on the Neuroimaging Standards for Research Into Small Vessel Disease (STRIVE) recommendation (Wardlaw et al., 2013).

Lacunes were defined as round or ovoid cerebrospinal fluid (CSF) containing cavities with a surrounding rim of hyperintensity in the subcortical regions and ranging from 3 to 15 mm in diameter. The severity of the periventricular and deep WMH was assessed using the Fazekas scale (Fazekas et al., 1987). CMBs were defined as rounded hypointense lesions (2–10 mm in diameter) on SWI sequences. EPVS were defined as round, ovoid, or linear lesions, generally smaller than 3 mm in diameter with a CSF-like signal on all sequences. The severity of EPVS was analyzed in the basal ganglia (BG) using a semi-quantitative scale (grade 0–4) (Doubal et al., 2010).

The total burden of the CSVD reflected the cumulative effect of the CSVD on brain damage. It was evaluated using the total CSVD score (from 0 to 4 points). If the imaging markers met any of the following criteria, one score was awarded: Fazekas score 3 for periventricular WMH or 2–3 for deep WMH, any lacune, any CMB, and EPVS score ≥ 2 in BG (Staals et al., 2014).

### Statistical Analysis

The data were analyzed using SPSS version 21.0 software (SPSS Inc., Chicago, IL, United States). The demographic variables of the participants are presented as means (standard deviation) or frequencies (percentage), when appropriate. One-way ANOVA or Chi-square test was used to compare the differences between the three groups (frail, prefrail, and robust). A multivariate linear regression analysis was performed to determine the relationships among the variables. The PROCESS macro for SPSS, developed by Hayes (Preacher and Hayes, 2004), was used to investigate the mediation effect of sleep quality in the relationship between CSVD burden and frailty. In all the analyses, age, sex, education level, HAMD score, and vascular risk factors were entered as covariates. A *P* < 0.05 was considered statistically significant.

## Results

**FIGURE 1:**
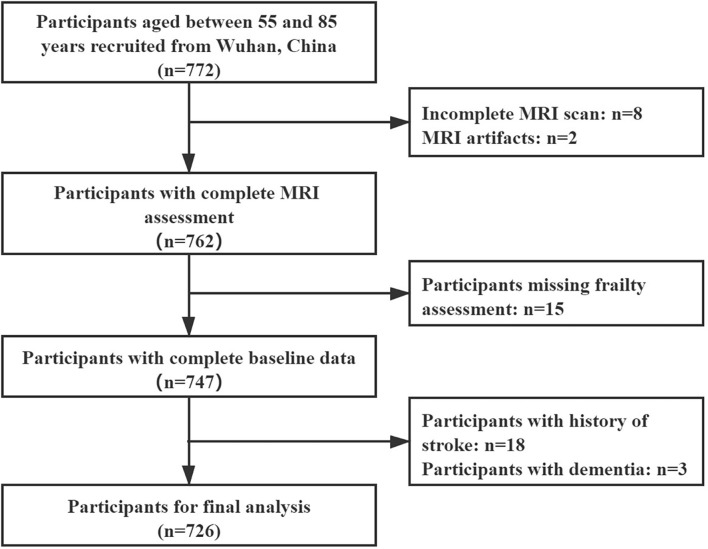
Participant inclusion flow chart. MRI, magnetic resonance imaging.

From November 2020 to May 2021, a total of 772 community residents participated in our study. Of the participants, ten of them were excluded due to failure to complete the MRI examination or having MRI artifacts. Fifteen residents were excluded due to a lack of data in the frailty assessment. Twenty-one residents were excluded due to a history of stroke or dementia. Finally, 726 residents were enrolled in the final analysis (Figure 1). Their characteristics are shown in Table 1. The mean age of the participants was 65.5 (±6.5) years. The majority of the participants were female (434, 59.8%). The numbers (percentages) of the frail, prefrail, and robust residents were 49 (6.7%), 310 (42.7%), and 367 (50.6%), respectively.

**TABLE 1:** Characteristics of the study participants according to frailty status.

Variables	Total (*n* = 726)	Frail (*n* = 49)	Prefrail (*n* = 310)	Robust (*n* = 367)	*P*
Age, mean (SD)	65.5 (6.5)	68.3 (6.2)	66.5 (6.9)	64.3 (5.9)	< 0.001
Female, *n* (%)	434 (59.8%)	29 (59.2%)	199 (64.2%)	206 (56.1%)	0.103
Education level	10.5 (3.8)	9.0 (4.3)	10.1 (3.9)	11.0 (3.5)	< 0.001
**Vascular risk factors**					
BMI, mean (SD)	23.8 (3.1)	24.2 (3.2)	23.9 (3.1)	23.7 (3.0)	0.458
Current smoker, *n* (%)	81 (11.2%)	5 (10.2%)	30 (9.7%)	46 (12.5%)	0.489
Diabetes, *n* (%)	136 (18.7%)	20 (40.8%)	61 (19.7%)	55 (15.0%)	< 0.001
Hypertension, *n* (%)	344 (47.4%)	27 (55.1%)	159 (51.3%)	158 (43.1%)	0.054
Hyperlipidemia, *n* (%)	185 (25.5%)	15 (30.6%)	83 (26.8%)	87 (23.7%)	0.458
HAMD*, mean (SD)	2.1 (1.7)	3.0 (1.9)	2.3 (1.8)	1.8 (1.4)	< 0.001
PSQI score, mean (SD)	6.9 (4.0)	11.6 (4.3)	8.2 (4.0)	5.2 (3.0)	< 0.001
**Neuroimaging Characteristics**					
CSVD burden, mean (SD)					< 0.001
0–1, *n* (%)	580 (79.9%)	21 (42.9%)	217 (70.0%)	342 (93.2%)	
2, *n* (%)	80 (11.0%)	11 (22.4%)	50 (16.1%)	19 (5.2%)	
3–4, *n* (%)	66 (9.1%)	17 (34.7%)	43 (13.9%)	6 (1.6%)	
Moderate-to-severe WMH, *n* (%)	192 (26.4%)	26 (53.1%)	117 (37.7%)	49 (13.4%)	< 0.001
Presence of lacunes, *n* (%)	113 (15.6%)	24 (49.0%)	67 (21.6%)	22 (6.0%)	< 0.001
Presence of CMBs, *n* (%)	118 (16.3%)	20 (40.8%)	61 (19.7%)	37 (10.1%)	< 0.001
>10 EPVS-BG, *n* (%)	150 (20.7%)	22 (44.9%)	88 (28.4%)	40 (10.9%)	< 0.001

*SD, standard deviation; BMI, body mass index; HAMD, Hamilton Depression Scale; PSQI, Pittsburgh Sleep Quality Index; CSVD, cerebral small vessel disease; WMH, white matter hyperintensities; CMBs, cerebral microbleeds; EPVS, enlarged perivascular spaces; BG, basal ganglia.*

*Excluding sleep item.

The frail residents tended to be older, with a lower education level, poorer sleep quality, higher score of HAMD, and a higher burden of CSVD. There were no significant differences in gender, body mass index (BMI), smoking habits, hypertension, and hyperlipidemia.

### Correlation Between the Key Variables

The results of the multivariate regression analysis are shown in Tables 2, 3. After adjusting for age, gender, education level, HAMD score, and vascular risk factors, the CSVD burden and PSQI score were associated with the frailty score, which indicates that participants with a higher CSVD burden or poorer sleep quality were more likely to be frail. In addition, a higher CSVD burden was also significantly correlated with poorer sleep quality. Additionally, the severity of the WMH, EPVS-BG, and the number of lacunes were correlated with frailty and sleep quality.

**TABLE 2:** Multivariate linear regression model with the frailty score as the dependent variable.

	Unadjusted model			Adjusted model*		
Frailty score	β	95% CI	*P*	β	95% CI	*P*
CSVD burden	0.43	0.36–0.50	< 0.001	0.41	0.34–0.48	< 0.001
Total WMH score	0.39	0.32–0.45	< 0.001	0.33	0.26–0.40	< 0.001
Number of lacunes	0.33	0.26–0.39	< 0.001	0.31	0.24–0.38	< 0.001
Number of CMBs	0.08	0.01–0.16	0.024	0.06	−0.01−0.13	0.100
EPVS-BG score	0.22	0.14−0.29	< 0.001	0.17	0.10−0.25	< 0.001
PSQI score	0.48	0.41−0.54	< 0.001	0.43	0.36−0.50	< 0.001

*CI, confidence interval; CSVD, cerebral small vessel disease; WMH, white matter hyperintensities; CMBs, cerebral microbleeds; EPVS, enlarged perivascular spaces; BG, basal ganglia; PSQI, Pittsburgh Sleep Quality Index.*

*Adjusted for age, gender, education level, HAMD score, and vascular risk factors.

**TABLE 3:** Multivariate linear regression model with the PSQI score as the dependent variable.

	Unadjusted model			Adjusted model*		
PSQI score	β	95% CI	*P*-value	B	95% CI	*P*
CSVD burden	0.24	0.17−0.32	< 0.001	0.25	0.18−0.32	< 0.001
Total WMH score	0.28	0.21−0.35	< 0.001	0.27	0.20−0.35	< 0.001
Number of lacunes	0.19	0.12−0.26	< 0.001	0.20	0.13−0.26	< 0.001
Number of CMBs	0.04	−0.03−0.11	0.292	0.04	−0.03−0.11	0.264
EPVS-BG score	0.11	0.04−0.18	0.003	0.12	0.05−0.19	0.001

*PSQI, Pittsburgh Sleep Quality Index; CI, confidence interval; CSVD, cerebral small vessel disease; WMH, white matter hyperintensities; CMBs, cerebral microbleeds; EPVS, enlarged perivascular spaces; BG, basal ganglia.*

*Adjusted for age, gender, education level, HAMD score, and vascular risk factors.

### Mediating Effect of Sleep Quality Between Cerebral Small Vessel Disease Burden and Frailty

**FIGURE 2:**
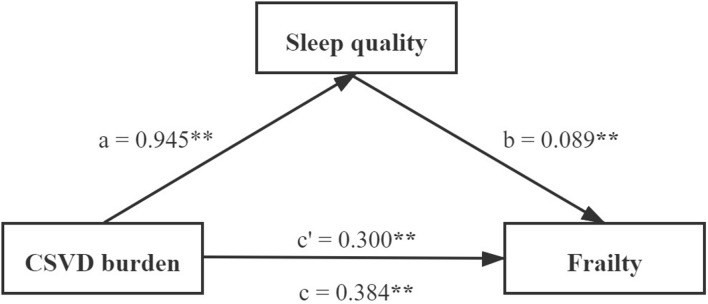
Path diagram of the association between CSVD burden and frailty with sleep quality as a mediator. **p < 0.001. Model control for age, gender, education level, HAMD score, and vascular risk factors. CSVD, cerebral small vessel disease.

Mediating effect analysis was performed using the PROCESS macro for SPSS (model four). The mediation pathway model with coefficients is shown in Figure 2. After adjusting for age, gender, education level, HAMD score, and vascular risk factors, the total effect (path c) and the direct effect (path c’) of the CSVD burden on frailty were significant (*c* = 0.384, *p* < 0.001; *c*’ = 0.300, *p* < 0.001). The indirect effect was 0.084 (path a × path b). The mediating effect of sleep quality was significant because 0 fell outside of the bootstrap 95% CI (Supplementary Table 3). Sleep quality played a partial mediating role in the association between CSVD burden and physical frailty. The mediating effect (ab/c) was 21.9%. In addition, Poor sleep quality mediated the relationships between frailty and imaging markers of CSVD (WMH, lacune, and EPVS-BG) (Supplementary Figure 1).

## Discussion

Our study was the first investigation to explore the mechanism underlying the association between CSVD burden and frailty. We discovered the role of sleep quality in mediating this association in community-dwelling older adults. Our results demonstrated that older people with a higher CSVD burden were more likely to be frail. This association was partially mediated by poor sleep quality.

In this study, the prevalence of frailty was 6.7% based on Fried’s criteria. Fried’s criteria is the most widely used definition for frailty assessments. This prevalence was similar to that found in a previous study that included 5,301 community-dwelling older adults from 28 Chinese cities (7.0%, based on Fried’s criteria) (Wu et al., 2017).

Although the existed associations between imaging markers of CSVD and frailty have been reported in the past, the results are inconsistent (Siejka et al., 2018, 2020; Kant et al., 2019; Maltais et al., 2019; Jordan et al., 2020). Differences in findings might be ascribed to the differences in the measurement of frailty in different study populations. In the present study, it was found that the severity of frailty was significantly related to the CSVD burden, the WMH score, the number of lacunes, and the EPVS-BG score, but not the number of CMBs.

Several previous studies have linked WMH to frailty (Siejka et al., 2018, 2020; Kant et al., 2019; Maltais et al., 2019; Sugimoto et al., 2019; Jordan et al., 2020). Sugimoto et al. (2019) and Siejka et al. (2020) reported that the severity of the baseline WMH was associated with the progression of frailty. WMH has also been linked to slow gait, falls, cognitive decline, and depression, which might increase the risk of frailty (de Leeuw et al., 2001; Srikanth et al., 2009). However, other studies only found a weak or no correlation between WMH and frailty (Chung et al., 2016; Kant et al., 2018). Interestingly, when the severity of WMH was assessed quantitatively, most previous studies reported positive correlations (Siejka et al., 2020). The link between frailty and lacunes is controversial due to the different definitions of lacunes or infarcts. For example, in three studies that used the criteria of STRIVE to assess lacunes (Chung et al., 2016; Siejka et al., 2018; Kant et al., 2019), only one study with the largest sample size (*n* = 962) showed a positive correlation between lacunes and frailty (Chung et al., 2016). No former studies displayed the relationship between frailty and EPVS severity, but it was found that high degree of EPVS-BG was positively correlated with the frailty in the present study. More large-size studies are required to confirm our findings.

Consistent with previous studies, our results showed that sleep quality was associated with CSVD and frailty (Del Brutto et al., 2015; Shih et al., 2020). Several previous community-based studies revealed that subjective sleep quality was associated with WMH and EPVS (Del Brutto et al., 2015, 2019; Sexton et al., 2017). The proportion of insomnia in elderly with CSVD was far higher than that among general elders (Wang et al., 2019). The comorbidity of CSVD and a sleep disorder might be related to chronic inflammation, oxidative stress, and other common pathways (Wang et al., 2020a,b). Further, several studies have demonstrated that people with poor sleep quality have increased odds of frailty (Pourmotabbed et al., 2020; Shih et al., 2020). A longitudinal study revealed that people with sleep disturbance at the baseline had a higher risk of frailty during the follow-up period (Ensrud et al., 2012). Sleep disorders might lead to frailty through dysregulation of various hormones (e.g., insulin-like growth factor-1 and testosterone), chronic inflammation, and other factors (Alvarez-Satta et al., 2020; Shih et al., 2020; Balomenos et al., 2021).

The crucial role of sleep quality in the development of frailty in individuals with higher CSVD burden was emphasized in this community-dwelling older adults cohort. It was supposed that individuals with a heavy burden of CSVD are more likely to have sleep disorders that subsequently lead to frailty through chronic inflammation, dysregulation of hormones, and other pathways (Balomenos et al., 2021). Targeted interventions toward sleep problems (e.g., medication, cognitive behavioral therapy, sleep hygiene education) (Kaur et al., 2020) could play a positive role in the prevention and treatment of frailty, thus reducing the incidence of adverse events.

Our study has a few limitations. First, this study was a cross-sectional study and thus could not establish a causal relationship between CSVD and frailty. Our findings should thus be interpreted with caution. In the future, interventional trials and basic research studies should be conducted to validate the causal relationship. Second, due to the low prevalence of frailty in the community population, the sample size of participants with frailty was small. Third, sleep quality was measured using a self-report instead of objective measures (e.g., polysomnography), which might have resulted in bias. Finally, our research subjects were Chinese-Asian people. Whether this result could be applied to non-Asian people is unclear and requires further research.

## Conclusion

Our study revealed that CSVD burden was associated with frailty among community-dwelling older adults. This association was partially mediated by sleep quality. Interventions targeted at sleep disorders might be a promising method to prevent physical frailty in CSVD patients.

## Data Availability Statement

The original contributions presented in the study are included in the article/Supplementary Material, further inquiries can be directed to the corresponding author.

## Ethics Statement

Written informed consent was obtained from the individual(s) for the publication of any potentially identifiable images or data included in this article.

## Author Contributions

JZ, XZ, YG, YZ, LW, and HH collected the clinical data. JZ, ZY, and WQ processed the statistical data. JZ drafted and revised the manuscript. XL designed and guided the study. All authors read and approved the final manuscript.

## Conflict of Interest

The authors declare that the research was conducted in the absence of any commercial or financial relationships that could be construed as a potential conflict of interest.

## Publisher’s Note

All claims expressed in this article are solely those of the authors and do not necessarily represent those of their affiliated organizations, or those of the publisher, the editors and the reviewers. Any product that may be evaluated in this article, or claim that may be made by its manufacturer, is not guaranteed or endorsed by the publisher.
