# Supplementary material for: Sleep Quality Mediates the Association Between Cerebral Small Vessel Disease Burden and Frailty: A Community-Based Study
Source: Front Aging Neurosci. 2021 Oct 20;13:751369. doi: 10.3389/fnagi.2021.751369 (PMC8564177; doi:10.3389/fnagi.2021.751369)
Supplement: Supplementary file 1 [file Table_1.DOCX]

***Supplementary Material***

**Supplemental Table 1: Frailty diagnosis criteria**

|  | male | female |
| --- | --- | --- |
| Weight loss | Baseline: ≥ 10 lbs (or 4.5kg) or ≥ 5% of original weight lost unintentionally in the prior year | |
| Slowness (4.57m) | Height ≤ 173cm, ≥ 7s  Height > 173cm, ≥ 6s | Height ≤ 159cm, ≥ 7s  Height > 159cm, ≥ 6s |
| Weakness(kg) | Grip strength  BMI ≤ 24kg/m^2^: ≤ 29  BMI 24.1-26.0 kg/m^2^: ≤ 30  BMI 26.1-28.0 kg/m^2^: ≤ 30  BMI > 28 kg/m^2^: ≤ 32 | Grip strength  BMI ≤ 23kg/m^2^: ≤ 17  BMI 23.1-26.0 kg/m^2^: ≤ 17.3  BMI 26.1-29.0 kg/m^2^: ≤ 18  BMI >29 kg/m^2^: ≤ 21 |
| Low activity* | < 383kcal/week | < 270kcal/week |
| Poor endurance;  Exhaustion | CES-D, Score 2-3 points for any question as follows:  How often over the past week they experienced symptoms?   1. I felt that everything I did was an effort. 2. I could not get “going.”   (0 =<1d; 1=1-2d; 2=3-4d; 3=>4d) | |

Notes: BMI = body mass index; CES-D = Center for Epidemiologic Studies Depression Scale.

* Physical activity was assessed by weekly caloric expenditure using the International Physical Activity Questionnaire short version

**Supplemental Table 2:** **Parameters of MRI sequences**

| MRI sequence | | Voxel size (mm) | FOV (mm) | TR (ms) | TE (ms) | Flip angle (deg) | Slice thickness (mm) |
| --- | --- | --- | --- | --- | --- | --- | --- |
| T1W | 1×1×1 | | 240×220 | 5.9 | 2.5 | 8 | 1 |
| T2W | 1×1×1 | | 240×220 | 3400 | 314.16 | NA | 1 |
| FLAIR | 1×1×1 | | 240×220 | 8000 | 518 | NA | 1 |
| SWI | 0.72×0.72×2 | | 230×230 | 30 | 20 | 15 | 2 |
| DWI | 1.44×1.44×3 | | 230×230 | 10113 | 73.6 | 90 | 3 |

Notes: MRI = magnetic resonance imaging; FOV = field of view; TR = repetition time; TE = echo time; T1W = T1-weighted; T2W = T2-weighted; FLAIR = fluid-attenuated inversion recovery; SWI = susceptibility-weighted imaging; DWI = diffusion-weighted image; NA = not available.

**Supplemental Table 3. Analysis of the mediating effect of sleep quality in the relationship between CSVD burden and frailty.**

|  |  |  | Bootstrap 95%CI | |  |
| --- | --- | --- | --- | --- | --- |
|  | β | Boot SE | Lower | Upper | PM(%) |
| Total effect | 0.38 | 0.03 | 0.32 | 0.45 | - |
| Direct effect | 0.30 | 0.03 | 0.24 | 0.36 | 78.1% |
| Indirect effect | 0.08 | 0.02 | 0.06 | 0.12 | 21.9% |

Notes: CSVD = cerebral small vessel disease; CI = confidence interval; SE = standard error; PM = percentage of mediation.


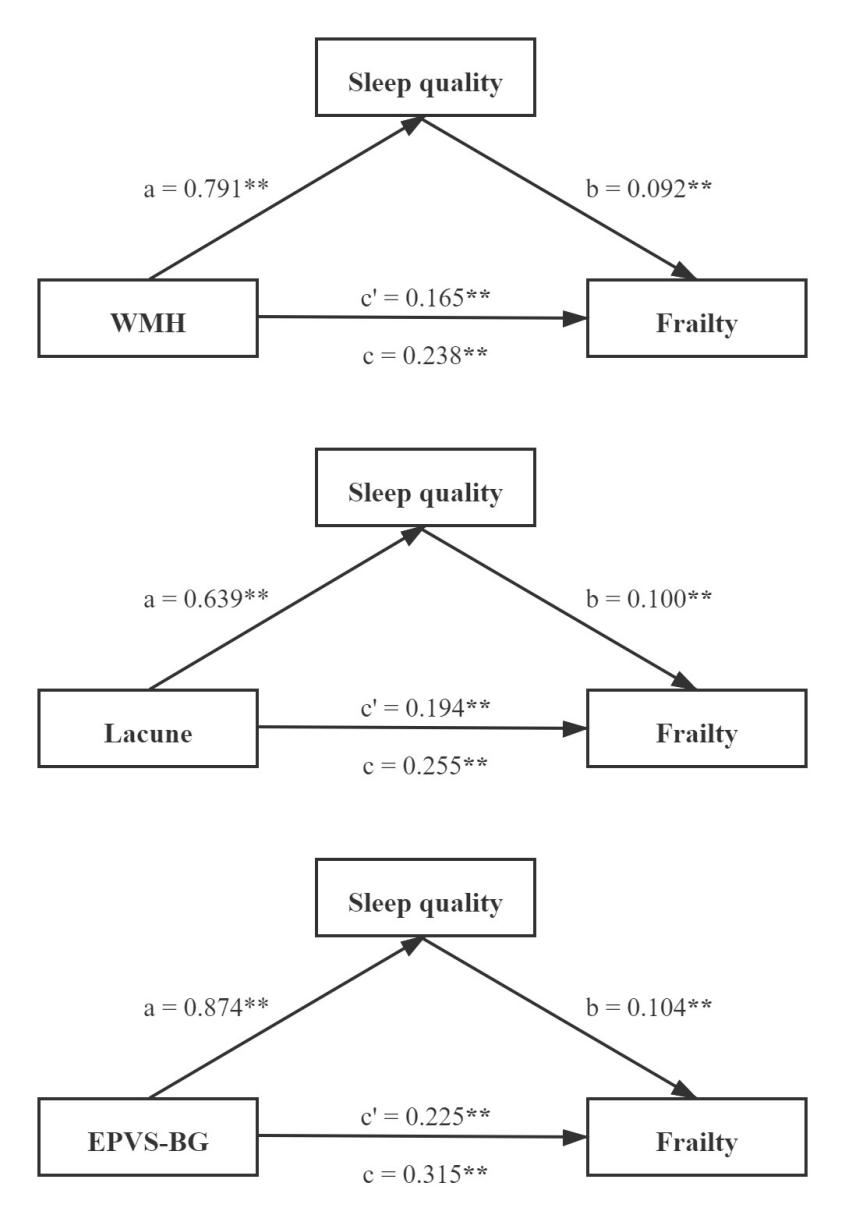


**Supplemental Figure 1: The mediation analysis by sleep quality of the association between imaging markers of CSVD and frailty.** ** p < 0.001. Each model included age, gender, education level, HAMD score, and vascular risk factors as covariates. WMH = white matter hyperintensities; EPVS = enlarged perivascular spaces; BG = basal ganglia.
